# Indirect Methods Produce Higher Estimates of Fine Root Production and Turnover Rates than Direct Methods

**DOI:** 10.1371/journal.pone.0048989

**Published:** 2012-11-09

**Authors:** Z. Y. Yuan, Han Y. H. Chen

**Affiliations:** Faculty of Natural Resources Management, Lakehead University, Thunder Bay, Ontario, Canada; Pennsylvania State University, United States of America

## Abstract

The production and turnover of fine roots play substantial roles in the biogeochemical cycles of terrestrial ecosystems. However, the disparity among the estimates of both production and turnover, particularly due to technical limitations, has been debated for several decades. Here, we conducted a meta-analysis to compare published estimates of fine root production and turnover rates derived from different methods at the same sites and at the same sampling time. On average, the estimates of fine root production and turnover rates were 87% and 124% higher, respectively, by indirect methods than by direct methods. The substantially higher fine root production and turnover estimated by indirect methods, on which most global carbon models are based, indicate the necessity of re-assessing the global carbon model predictions for atmospheric carbon sequestration in soils as a result of the production and turnover of fine roots.

## Introduction

Although fine roots represent a small component of total vegetation biomass, fine root production and turnover are major contributors to carbon (C) and nutrient cycles [1]–[4]. Current estimates indicate that fine root production might contribute as much as 77% of the total net primary production in forest ecosystems [1], [5]–[8]. At the global scale, fine root turnover is estimated to transfer 33% of annual net primary production to the soil in terrestrial ecosystems [1]. Fine root production and turnover vary widely both within and among species and ecosystems [9]–[14] and may be sensitive to changing climate and soil environments [15], [16]. Fine root production and turnover are key processes in ecosystem carbon models, e.g., CENTURY [17] and Biome-BGC [18], and most ecosystem carbon models are based on indirect estimates of fine root production and turnover. Accurate estimates of fine root production and turnover rates, however, have been elusive. Even at a single site and measured at the same year, estimates of fine root production and turnover rates may vary greatly depending upon the methods used [19]–[22]. The variations in fine root production and turnover rates in published literature, therefore, could either reflect differences driven by ecological processes or methodological differences. However, the extent of such methodological differences remains poorly understood.

Various methods have been used to study root systems. The existing methods can be classified into two groups: direct and indirect [20], [23], both of which have strengths and limitations [24]–[26]. Direct methods to estimate fine root production include ingrowth cores and minirhizotrons, while indirect methods include sequential coring, nitrogen (N) budget, C budget, stable or radiocarbon isotopic method, and correlations with abiotic resources [20], [27], [28]. Among all methods for estimating fine root production, the use of minirhizotrons, albeit requiring some assumptions, is a technique that allows the tracking of fine root growth/death. Thus, it is generally considered as a more ‘direct’ method for observing fine root turnover directly through a rhizotron or root observation window [23], [29].

Of all methods, sequential soil coring has been the most commonly used in published literature. This method measures the changes of living and/or dead root biomass sampled during a period of one year or longer. From the sequential biomass data, fine root production and turnover rates are indirectly estimated by: 1) “maximum-minimum” (hereafter max-min) method based on differences in biomass between the maximum and minimum fine root biomass measured during a year, 2) “decision matrix” method or “balancing transfer” calculations [30] based on the variation in the biomass and the necromass between successive sampling dates, 3) “sum of changes” based on all positive differences in biomass/necromass between successive sampling dates, or 4) “compartment-flow model” technique [5], which takes into account of the decomposition rates of dead fine roots and includes two compartments (live and dead) and three flows (production, mortality and decomposition). The method of “sum of changes” can be further divided into two approaches, i.e., summing all positive changes or only statistically significant increment of fine root biomass.

Although estimates of fine root production and turnover have been reported to vary between direct and indirect estimates [14], [20], [26], [31], the relationships between those two estimates are still unclear because most studies refer only to one or two stands and contradictory results are often found between studies. Further generalization is needed to clarify how and to what extent indirect estimates are related to direct estimates. In this paper, our central question was whether commonly used fine root sampling methods resulted in similar estimates of fine root production and turnover. Because fine root production and turnover rates vary greatly in response to spatial and temporal variations [20], [31], we only selected paired data collected from the same sites and sampling dates to examine the estimates among different methods.

## Methods

Data on fine root production and turnover rates were searched from published studies (Supporting Material S1) using journal search tools (Web of Science, PubMed, JSTOR, and Google Scholar). Fine roots are typically assumed to be roots <2 mm in diameter [3]. Data for estimating fine root production (Mg ha^−1^ year^−1^) were derived from eleven methods: ingrowth, minirhizotrons, max-min, decision matrix, sum of all positive changes, sum of significant positive changes, compartment-flow model, N budget, C budget, isotopic and correlation methods. Based on previous criteria [20], [32], ingrowth and minirhizotrons are considered as direct methods, and the rest are indirect methods for estimating fine root production. The methods of max-min, decision matrix, sum of changes, and compartment-flow model derived from sequential soil cores were considered as indirect methods because they estimated fine root production indirectly from biomass data [32].

In our paper, we only considered minirhizotron technique as a direct method for estimating fine root turnover (year^−1^), i.e., the fraction of a root system that is renovated during a year through the replacement by new root growth for the death of some roots, because it can provide direct observations for root dynamics through time. Fine root turnover from indirect methods was defined as the ratio of the total amount of live fine roots produced in one year (Mg ha^−1^ year^−1^) over the mean standing biomass (Mg ha^−1^) of fine roots [21]. When turnover coefficients, longevity (or lifespan, turnover time), and/or turnover index were reported, fine root turnover rates were calculated accordingly.

Only paired data (means with standard deviations for direct and indirect cases) collected from the same sites, sampling dates and layers were selected in the data set, which encompassed 45 sources from 120 sites. Among them, there were 20 studies in which multiple observations from different sampling dates/layers at the same site were reported. In order to conduct analysis with independent observations, we only used the observations from the first sampling dates/layers in our analysis.

All original data were extracted from the text, tables and figures in the published literature. We used standard deviations reported in the original studies or calculated standard deviations from the standard error and the number of replicates. Studies that did not report standard error or deviation were not included in the data set. When data were presented graphically, numerical data were obtained by using Image-Pro Plus 7.0 (Media Cybernetics, Inc., MD).

Since fine root production and turnover rates were not normally distributed, they were transformed by logarithm (base 10) to better achieve normality (Shapiro-Wilk test) and homogeneous variances (Levene test). The difference between the average estimates among methods was tested by one-way analysis of variance. Model II regression analysis in package *lmodel2* was used to evaluate the correlations between estimates of fine root production and turnover rates for each method.

To examine whether estimates from indirect methods were significantly different from direct methods, we calculated effect sizes from each individual study as described by Hedges et al. [33]. Effect size was calculated as a natural log response ratio: ln*RR = *ln (*X_e_*/*X_c_*) = ln *X_e_* - ln *X_c_*, where *X_e_* and *X_c_* were mean fine root production or turnover by indirect and direct methods, respectively. The Q statistic was calculated to test the homogeneity between studies. A large value of Q indicates significant heterogeneity between studies. To take heteroscedastic sampling variances among individual studies into account, we calculated the corresponding sampling variance for each ln*RR* as ln[(1/*n*_e_)×(*S*_e_/*X*_e_)^2^+(1/*n*_c_)×(*S*_c_/*X*_c_)^2^] in package *metafor* 1.60 for R [34], [35], where *n*_e_, *n*_c_ *S*_e_, *S*_c_ *X_e_*, and *X_c_* are sample sizes, stand deviations, and means of fine root production/turnover by indirect and direct methods, respectively.

To examine if the difference in production between direct and indirect estimates, where a sufficient number of paired observations are available (*n* = 214), is consistent among indirect methods or biomes, we used mixed-random effect models by including indirect method or biome as a fixed factor [34], [35]. Because the number of turnover estimates is small (*n* = 21), effect size was only analyzed without considering potential difference among indirect methods or biomes. The difference between direct and indirect estimates was considered significant if the 95% confidence interval (CI) of *RR* did not overlap 1. All statistical analyses were performed in R 2.14.1.

## Results

**Figure 1:**
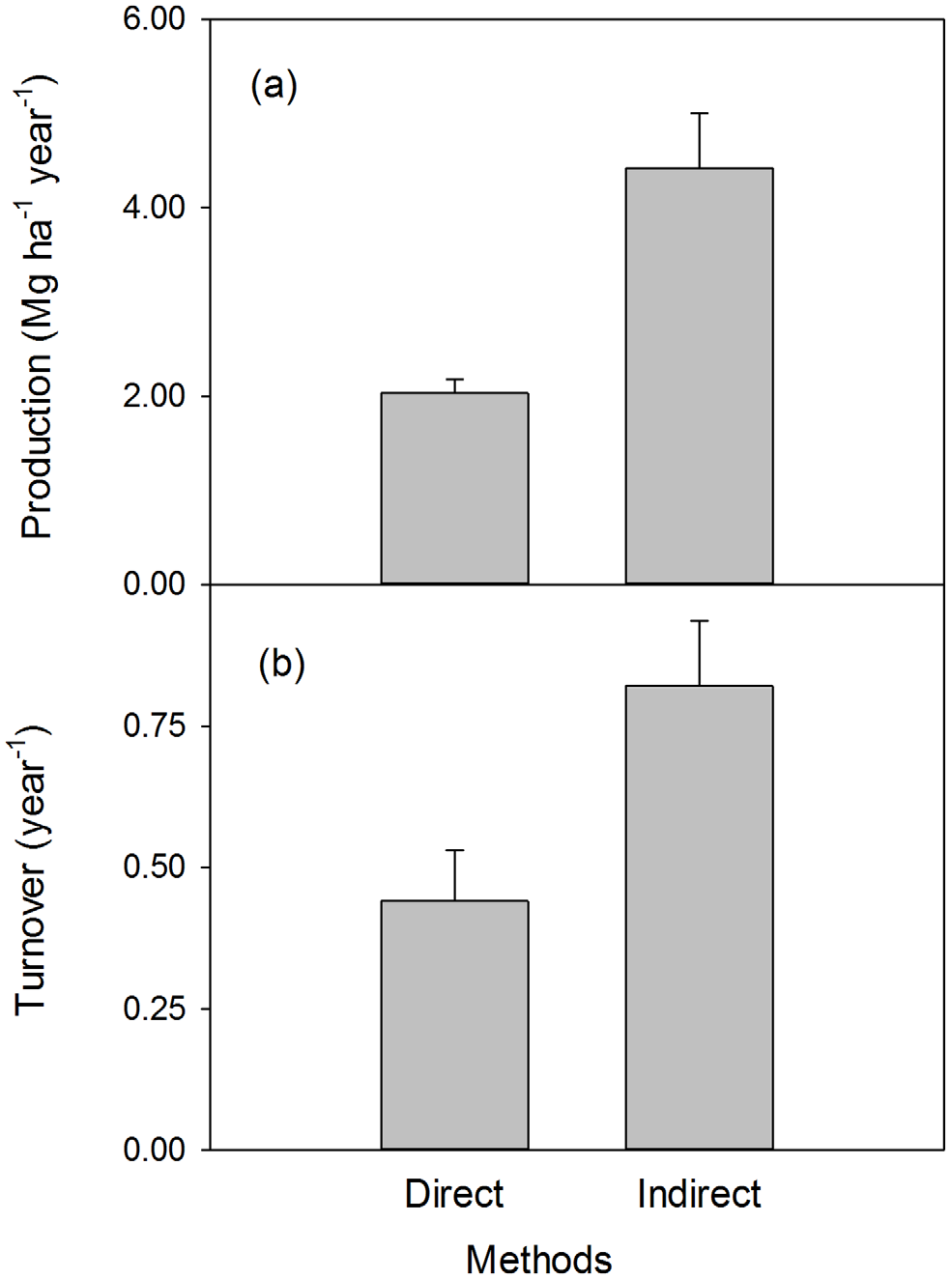
Differences between direct and indirect estimates. (a) fine root production and (b) turnover rates. Estimates (mean±1 SE) are derived from the same sites sampled in the same year: Both *P*<0.05 between direct and indirect estimates.

**Figure 2:**
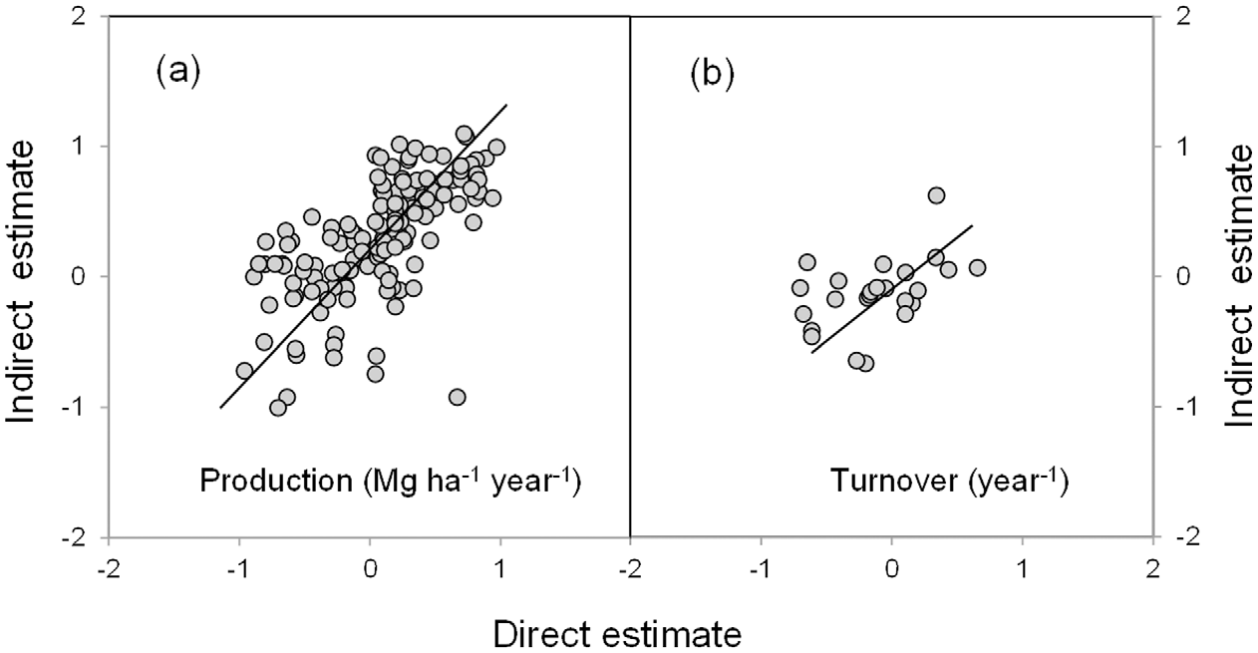
Relationships of direct estimates to indirect estimates. (a) fine root production and (b) turnover rates. The values of fine root production (Mg ha^−1^ year^−1^) and turnover rates (year^−1^) are log-transformed (base 10). The relationships are best described by linear regressions (production: log_10_ *y* = 0.253+0.632×log_10_ *x*, *r* ^2^ = 0.418, *P*<0.001; turnover rates: log_10_ *y* = 0.089+0.324×log_10_ *x*, *r* ^2^ = 0.207, *P*<0.05).

Indirect methods yielded significantly higher values of both fine root production and turnover than the direct methods (Fig. 1, *P*<0.001). When data from direct and indirect methods were respectively pooled, the estimates of fine root production were significantly correlated between direct and indirect methods (Fig. 2a). Similarly, there was a significantly positive correlation between direct and indirect estimates of fine root turnover rates (Fig. 2b).

**Figure 3:**
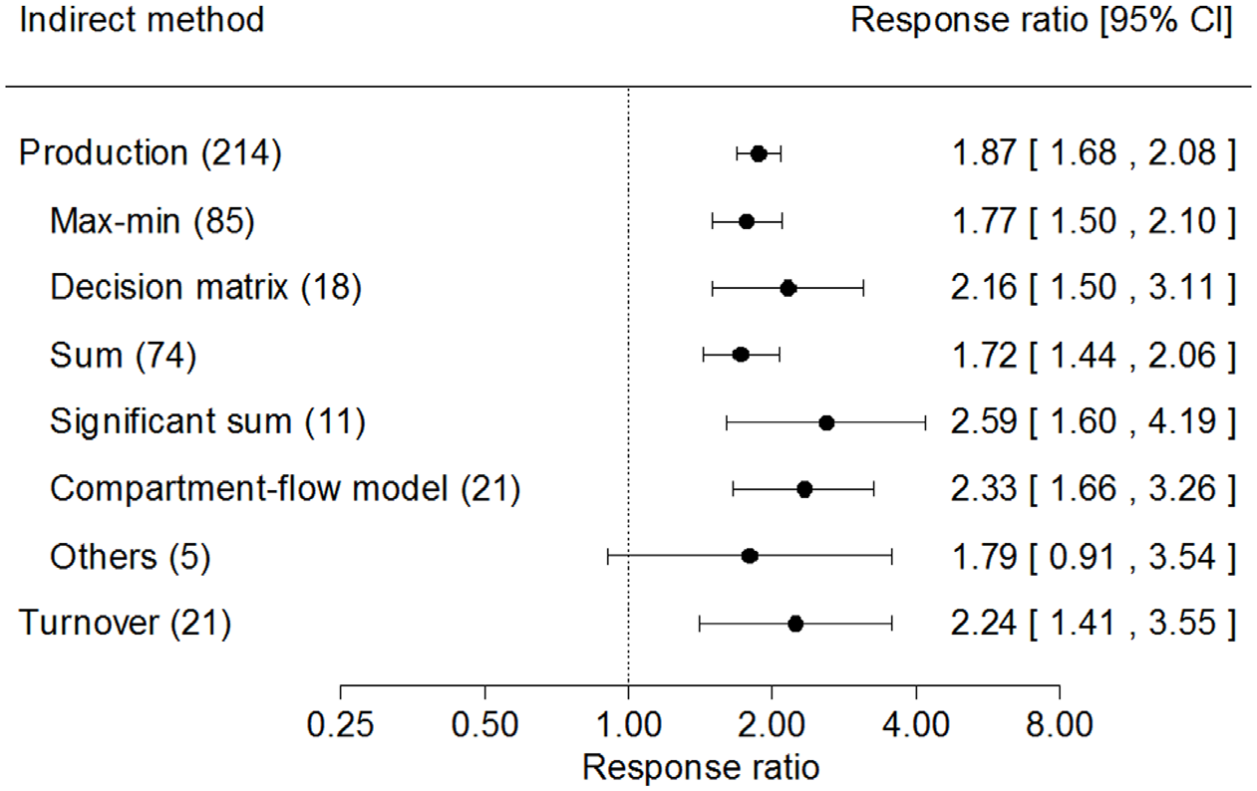
Response ratios for each indirect method. The number in parentheses represents the number of observations. The dot with error bars shows the mean effect size with the 95% confidence interval. The methods of ‘others’ refer to carbon and correlation methods.

Overall, the Q statistic values for fine root production and turnover were 87984 and 1003, respectively. Both *P* values were less than 0.0001, indicating significant heterogeneity between studies. By using balanced data from the same site and same sampling time in meta-analysis, the random effect model showed significant differences between direct and indirect methods both in fine root production and in turnover (*P*<0.001 and <0.01, respectively) (Fig. 3). With all data pooled, fine root production estimated from indirect methods was 87% higher than from direct methods. The mean response ratios were significantly higher than 1.00 for indirect methods of max-min (*RR = *1.77), decision matrix (*RR = *2.16), summing all positive increment (*RR = *1.72), summing significantly positive increment (*RR = *2.59), compartment-flow models (*RR* = 2.33) (all *P*<0.001), but not significantly different from 1.00 for other methods (*RR = *1.79, *P* = 0.140). The differences in response ratios among indirect estimates for fine root production were not significant. Fine root turnover estimated from indirect methods was 2.24 times higher than from direct methods and significantly higher than 1.00 (*P*<0.01) (Fig. 3).

**Figure 4:**
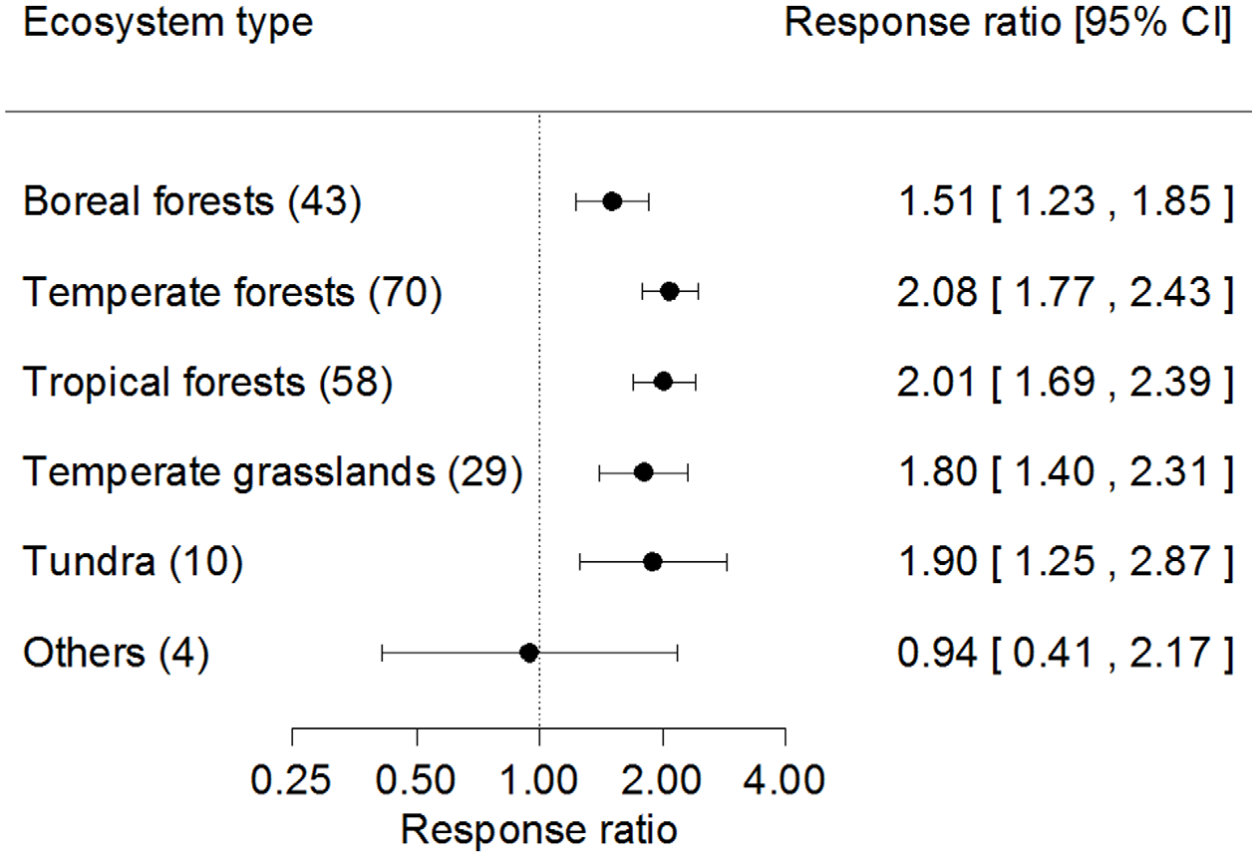
Response ratios for fine root production among ecosystem types. The number in parentheses represents the number of observations. The dot with error bars shows the mean effect size with the 95% confidence interval. The ecosystem of ‘others’ refers to deserts and wetlands.

Among biomes, the estimates for fine root production from indirect methods were greater than from direct methods in most biomes, except in deserts and wetlands (Fig. 4). The mean response ratios were significantly higher than 1.00 for indirect methods in boreal forests (*RR = *1.51), temperate forests (*RR = *2.08), tropical forests (*RR = *2.01), temperate grasslands (*RR = *1.80), and tundra (*RR* = 1.90) (all *P*<0.01), but not significantly different from 1.00 for indirect methods in other biomes (including deserts and wetlands) (*RR = *0.94, *P* = 0.931). There were no significant differences in response ratios for fine root production between biomes.

## Discussion

Studies that used more than one method to estimate fine root production and turnover rates from the same site and sampling time, allowing for the control of spatial and temporal variability in belowground processes, present useful insights into methodological differences in estimating fine root production and turnover rates. In our meta-analysis, significant differences were found in fine root production and turnover between direct and indirect methods, with higher estimates in both production and turnover by indirect methods than by direct methods. Together with the log-intercept in the correlations between indirect and direct estimates, especially at the low end (log-intercept >0 for both production and turnover rates), our analysis suggested that indirect methods generally yielded higher estimates of fine root production and turnover rates. The consistent relationships between direct and indirect methods found in our meta-analysis have advanced the understanding of the consequences of methodological differences [10]. Both direct and indirect methods have limitations in estimating or measuring fine root production. For example, both direct methods such as ingrowth cores and minirhizotrons that create soil disturbances can either reduce or stimulate fine root production, whereas indirect methods are inherently based on assumptions that are specific to each method [10]. Therefore, our findings cannot be used to infer which method can produce the ‘true’ measure of fine root production, and multiple methods are still recommended for yielding realistic estimates of fine root production [31]. Nevertheless, since most global ecosystem carbon models are based on the consistently higher estimates from indirect methods, the predictions for CO2 sequestration in soils through fine root production and turnover from these models may need to be reassessed.

The estimates of fine root production were consistent among indirect methods resulted from sequential coring data. Incorporating not only significant but also non-significant differences of random errors into the estimates could be one of the reasons for the higher estimates of fine root production by max-min, decision matrix, sum of changes, and compartment-flow methods. However, summing only significant increment of fine root biomass also produced higher estimates than direct methods, but the reasons were unclear. Despite higher estimates from sequential core data than direct methods, unless enough samples can capture all seasonal minima and maxima of fine root dynamics, sequential soil coring can yield underestimation of production.

Due to the small size of other indirect methods (*n* = 5), it remained unclear whether C budget, N budget, isotopic and correlation methods generally yielded significantly higher estimates than direct methods. Studies employing the sequential core techniques have found that sequential soil core-based methods such as max-min and decision matrix yield low production estimates compared to non-soil core methods [31], [36]. In our analysis, however, the estimates from sequential core techniques were not significantly different from other non-soil core methods, again likely reflecting the small size of non-soil core methods.

Our meta-analysis showed that indirect methods also generally yielded higher estimates of fine root turnover rates by more than two times of those from direct method, *i.e.*, minirhizotron technique (Fig. 3). Because mass-based turnover estimates of fine roots are generally derived from production (flux) and biomass (pool), it was not surprising to find similar patterns of fine root production and turnover between direct and indirect methods. This was also reasonable as production generally increases with increasing turnover rates. However, the limited number of paired observations in our study for fine root turnover between direct and indirect methods (*n* = 21) suggests that further investigations are needed to examine the difference in turnover estimates and make a clear conclusion. Despite the higher estimates from all indirect methods, sequential soil coring is often criticized for its failure to capture all fine root dynamics and C&N budget methods generally cannot estimate nutrient fluxes accurately in natural ecosystems, consequently underestimating fine root turnover rates. Therefore, indirect methods should be used with caution unless all seasonal minima & maxima of fine root dynamics or resource pools and fluxes can be accurately determined.

Overall, our meta-analysis showed the estimates of both fine root production and turnover rates were on average higher by indirect than direct methods, and found positive correlations between estimates from indirect and direct methods. However, there is no possibility of testing whether either of these methods can produce true estimates of fine root production and turnover in natural environments. It is possible that both indirect and direct methods underestimate fine root production and turnover. If direct methods yield estimates that are closer to the ‘true’ values, the higher fine root production and turnover estimates by indirect methods, on which most global carbon models are based, indicate the necessity to re-assess the extent to which atmospheric carbon sequestration in soils.

## Supporting Information

Supporting Material S1 List of studies included in the meta-analysis.
(PDF)
