# Supplementary material for: Indirect Methods Produce Higher Estimates of Fine Root Production and Turnover Rates than Direct Methods
Source: PLoS One. 2012 Nov 9;7(11):e48989. doi: 10.1371/journal.pone.0048989 (PMC3494664; doi:10.1371/journal.pone.0048989)
Supplement: Supporting Material S1 — List of studies included in the meta-analysis. (PDF) [file pone.0048989.s001.pdf]

Supporting material

Studies included in the meta-analysis:

Adamek, M. 2009. Effects of increased nitrogen input on the net primary production of a tropical lower montane rain foest, Panama. Niedersahsischen Staats-und Universitat sbibliothek Gottingen.

Adamek, M., M. D. Corre, and D. Holscher. 2011. Responses of fine roots to experimental nitrogen addition in a tropical lower montane rain forest, Panama. Journal of Tropical Ecology **27**:73-81.

Boxman, A. W., K. Blanck, T. E. Brandrud, B. A. Emmett, P. Gundersen, R. F. Hogervorst, O. J. Kjonaas, H. Persson, and V. Timmermann. 1998. Vegetation and soil biota response to experimentally-changed nitrogen inputs in coniferous forest ecosystems of the NITREX project. Forest Ecology and Management **101**:65-79.

Brassard, B. W., H. Y. H. Chen, Y. Bergeron, and D. Pare. 2011. Differences in fine root productivity between mixed- and single-species stands. Functional Ecology **25**:238-246.

Brunner, I., E. G. Pannatier, B. Frey, A. Rigling, W. Landolt, S. Zimmermann, and M. Dobbertin. 2009. Morphological and physiological responses of Scots pine fine roots to water supply in a dry climatic region in Switzerland. Tree Physiology **29**:541-550.

Davis, J. P., B. Haines, D. Coleman, and R. Hendrick. 2004. Fine root dynamics along an elevational gradient in the southern Appalachian Mountains, USA. Forest Ecology and Management **187**:19-34.

Dress, W. J. and R. E. J. Boerner. 2001. Root dynamics of southern Ohio oak-hickory forests: influences of prescribed fire and landscape position. Canadian Journal of Forest Research **31**:644-653.

Fang, Q. L. and L. Q. Sha. 2005. Study of fine roots biomass and turnover in the rubber plantation of Xishuangbanna. Journal of Central South Forestry University **25**:40-44.

Finer, L. and J. Laine. 2000. The ingrowth bag method in measuring root production on peatland sites. Scandinavian Journal of Forest Research **15**:75-80.

Fisk, M. C., S. K. Schmidt, and T. R. Seastedt. 1998. Topographic patterns of above- and belowground production and nitrogen cycling in Alpine tundra. Ecology **79**:2253-2266.

Gao, Y. Z., M. Giese, S. Lin, B. Sattelmacher, Y. Zhao, and H. Brueck. 2008. Belowground net primary productivity and biomass allocation of a grassland in Inner Mongolia is affected by grazing intensity. Plant and Soil **307**:41-50.

Garcia-Pausas, J., P. Casals, J. Romanya, S. Vallecillo, and M. T. Sebastia. 2011. Seasonal patterns of belowground biomass and productivity in mountain grasslands in the Pyrenees. Plant and Soil **340**:315-326.

Gaul, D., D. Hertel, W. Borken, E. Matzner, and C. Leuschner. 2008a. Effects of experimental drought on the fine root system of mature Norway spruce. Forest Ecology and Management **256**:1151-1159.

Gaul, D., D. Hertel, and C. Leuschner. 2008b. Effects of experimental soil frost on the fine-root system of mature Norway spruce. Journal of Plant Nutrition and Soil Science-Zeitschrift Fur Pflanzenernahrung Und Bodenkunde **171**:690-698.

Gaul, D., D. Hertel, and C. Leuschner. 2009. Estimating fine root longevity in a temperate Norway spruce forest using three independent methods. Functional Plant Biology **36**:11-19.

Handa, I. T., F. Hagedorn, and S. Hattenschwiler. 2008. No stimulation in root production in response to 4 years of in situ CO_2 enrichment at the Swiss treeline. Functional Ecology **22**:348-358.

Hansson, A. C. and O. Andren. 1986. Belowground plant-production in a perennial grass ley (*Festuca pratensis* Huds) assessed with different methods. Journal of Applied Ecology **23**:657-666.

Hendricks, J. J., R. L. Hendrick, C. A. Wilson, R. J. Mitchell, S. D. Pecot, and D. L. Guo. 2006. Assessing the patterns and controls of fine root dynamics: An empirical test and methodological review. Journal of Ecology **94**:40-57.

Hertel, D., M. A. Harteveld, and C. Leuschner. 2009. Conversion of a tropical forest into agroforest alters the fine root-related carbon flux to the soil. Soil Biology & Biochemistry **41**:481-490.

Joslin, J. D. and G. S. Henderson. 1987. Organic-matter and nutrients associated with fine root turnover in a white oak stand. Forest Science **33**:330-346.

Kiley, D. K. and R. L. Schneider. 2005. Riparian roots through time, space and disturbance. Plant and Soil **269**:259-272.

Langley, J. A., P. Dijkstra, B. G. Drake, and B. A. Hungate. 2003. Ectomycorrhizal colonization, biomass, and production in a regenerating scrub oak forest in response to elevated CO_2. Ecosystems **6**:424-430.

Li, J. Z., S. Lin, F. Taube, Q. M. Pan, and K. Dittert. 2011. Above and belowground net primary productivity of grassland influenced by supplemental water and nitrogen in Inner Mongolia. Plant and Soil **340**:253-264.

Lukac, M., C. Calfapietra, and D. L. Godbold. 2003. Production, turnover and mycorrhizal colonization of root systems of three Populus species grown under elevated CO^2 (POPFACE). Global Change Biology **9**:838-848.

Lukac, M. and D. L. Godbold. 2010. Fine root biomass and turnover in southern taiga estimated by root inclusion nets. Plant and Soil **331**:505-513.

Marchesini, L. B., D. Papale, M. Reichstein, N. Vuichard, N. Tchebakova, and R. Valentini. 2007. Carbon balance assessment of a natural steppe of southern Siberia by multiple constraint approach. Biogeosciences **4**:581-595.

Mei, L., J. C. Gu, Z. W. Zhang, and Z. Q. Wang. 2010. Responses of fine root mass, length, production and turnover to soil nitrogen fertilization in *Larix gmelinii* and *Fraxinus mandshurica* forests in Northeastern China. Journal of Forest Research **15**:194-201.

Meinen, C., D. Hertel, and C. Leuschner. 2009. Root growth and recovery in temperate broad-Leaved forest stands differing in tree species diversity. Ecosystems **12**:1103-1116.

Metcalfe, D. B., P. Meir, and M. Williams. 2007. A comparison of methods for converting rhizotron root length measurements into estimates of root mass production per unit ground area. Plant and Soil **301**:279-288.

Moser, G., C. Leuschner, M. Roderstein, S. Graefe, N. Soethe, and D. Hertel. 2010. Biomass and productivity of fine and coarse roots in five tropical mountain forests stands along an altitudinal transect in southern Ecuador. Plant Ecology & Diversity **3**:151-164.

Neill, C. 1992. Comparison of soil coring and ingrowth methods for measuring belowground production. Ecology **73**:1918-1921.

Neill, C. 1994. Primary production and management of seasonally flooded prairie marshes harvested for wild hay. Canadian Journal of Botany **72**:801-807.

Pei, Z. Q., Y. Zhou, Y. R. Zheng, and C. W. Xiao. 2011. Contribution of fine root turnover to the soil organic carbon cycling of *Reaumuria soongorica* community in an arid ecosystem. Chinese Journal of Plant Ecology **35**:0-0.

Persson, H. 1979. Fine-root production, mortality and decomposition in forest ecosystems. Vegetatio **41**:101-109.

Persson, H. A. 1983. The distribution and productivity of fine roots in boreal forests. Plant and Soil **71**:87-101.

Powell, S. W. and F. P. Day. 1991. Root production in four communities in the Great Dismal Swamp. American Journal of Botany **78**:288-297.

Pridoehl, F. 2010. Fine root dynamics for three distinct northern Ontario forests: A comparison of approaches used to estimate fine root biomass, productivity, and turnover. Lakehead University, ON.

Rodriguez, M. V., M. B. Bertiller, and A. Bisigato. 2007. Are fine roots of both shrubs and perennial grasses able to occupy the upper soil layer? A case study in the arid Patagonian Monte with non-seasonal precipitation. Plant and Soil **300**:281-288.

Sanford, R. L. 1990. Fine root biomass under light gap openings in an Amazon rain forest. Oecologia **83**:541-545.

Singh, B. 1998. Contribution of forest fine roots in reclamation of semiarid sodic soils. Arid Soil Research and Rehabilitation **12**:207-222.

Sun, Y., J. Gu, H. Zhuang, D. Guo, and Z. Wang. 2011. Lower order roots more palatable to herbivores: a case study with two temperate tree species. Plant and Soil **347**:351-361.

Symbula, M. and F. P. Day. 1988. Evaluation of two methods for estimating belowground production in a fresh-water swamp forest. American Midland Naturalist **120**:405-450.

Tripathi, S. K., A. Sumida, H. Shibata, S. Uemura, K. Ono, and T. Hara. 2005. Growth and substrate quality of fine root and soil nitrogen availability in a young Betula ermanii forest of northern Japan: Effects of the removal of understory dwarf bamboo (*Sasa kurilensis*). Forest Ecology and Management **212**:278-290.

Wang, W., J. Feng, and T. Oikawa. 2009. Contribution of root and microbial respiration to soil CO_2 efflux and their environmental controls in a humid temperate grassland of Japan. Pedosphere **19**:31-39.

Wu, Y. B., J. Wu, Y. C. Deng, H. C. Tan, Y. G. Du, S. Gu, Y. H. Tang, and X. Y. Cui. 2011. Comprehensive assessments of root biomass and production in a Kobresia humilis meadow on the Qinghai-Tibetan Plateau. Plant and Soil **338**:497-510.
